# Crystal structure of 1-[(4-chloro­phen­yl)di­phenylyl­meth­yl]-3-(tri­fluoro­meth­yl)-1*H*-pyrazole

**DOI:** 10.1107/S2056989025001185

**Published:** 2025-02-18

**Authors:** Firudin I. Guseinov, Ksenia A. Afanaseva, Vera A.Vil, Bogdan I. Ugrak, Aida I. Samigullina, Ennio Zangrando, Alebel N. Belay

**Affiliations:** a Kosygin State University of Russia, 117997 Moscow, Russian Federation; b N. D. Zelinsky Institute of Organic Chemistry, Russian Academy of Sciences, 119991 Moscow, Russian Federation; c Department of Chemical and Pharmaceutical Sciences, University of Trieste, 34127, Trieste, Italy; d Department of Chemistry, Bahir Dar University, PO Box 79, Bahir Dar, Ethiopia; University of Durham, United Kingdom

**Keywords:** crystal structure, tri­fluoro­methyl­pyrazole, tri­fluoro­meth­yl, non-covalent inter­actions

## Abstract

The title compound, C_23_H_16_ClF_3_N_2_, was synthesized from 3-(tri­fluoro­meth­yl)-1*H*-pyrazole and chloro­(4-chloro­phen­yl)methyl­ene)di­benzene. The structure features intra­molecular (Ph)C—H⋯N and inter­molecular (Ph)C—H⋯F hydrogen bonds, as well as C—H⋯π-ring inter­actions between the phenyl and pyrazole rings.

## Abstract 2

The title pyrazole derivative, which exhibits multiple inter­molecular non-covalent inter­actions, was synthesized by the reaction of 3-(tri­fluoro­meth­yl)-1*H*-pyrazole with chloro­(4-chloro­phen­yl)methyl­ene)di­benzene in the presence of K_2_CO_3_ in tetra­hydro­furan.

## 1. Chemical context

Pyrazoles and their derivatives are found in natural compounds and drugs, and are widely used in organic synthesis (Guseinov *et al.*, 2006 ▸, 2024 ▸; Küçükgüzel *et al.*, 2015 ▸; Pizzuti *et al.*, 2014 ▸). Similarly to hydrazones (Mahmudov *et al.*, 2011 ▸), new pyrazole derivatives can also be used in crystal engineering as well as in the synthesis of coordination compounds for catalysis (Jlassi *et al.*, 2014 ▸; Ma *et al.*, 2021 ▸; Mac Leod *et al.*, 2012 ▸) and biological studies (Martins *et al.*, 2017 ▸). The hydrogen-bond acceptor ability of the pyrazole motif can be employed as a tool for crystal growth and design (Guseinov *et al.*, 2017 ▸, 2022 ▸; Abdelhamid *et al.*, 2011 ▸; Afkhami *et al.*, 2017 ▸). We believe that the attachment of a tri­fluoro­methyl group to the pyrazole ring can improve the functional properties of new derivative ligands or supra­molecular synthons. In fact, tri­fluoro­methyl­ated pyrazoles are indispensable heterocyclic motifs that constitute the core of a variety of bioactive substrates and pharmaceuticals (Kumar *et al.*, 2023 ▸; Westphal *et al.*, 2015 ▸; Zhu *et al.*, 2014 ▸). Among them, the 3-tri­fluoro­methyl­pyrazole scaffold is of great medicinal significance and is present in several drugs and bioactive mol­ecules including celecoxib, mavacoxib (anti-inflammatory), razaxaban (anti­coagulant), SC-560 (anti­tumor) and penthio­pyrad (anti­fungal) (Davis *et al.*, 2013 ▸; Fang *et al.*, 2020 ▸; Liu *et al.*, 2023 ▸).
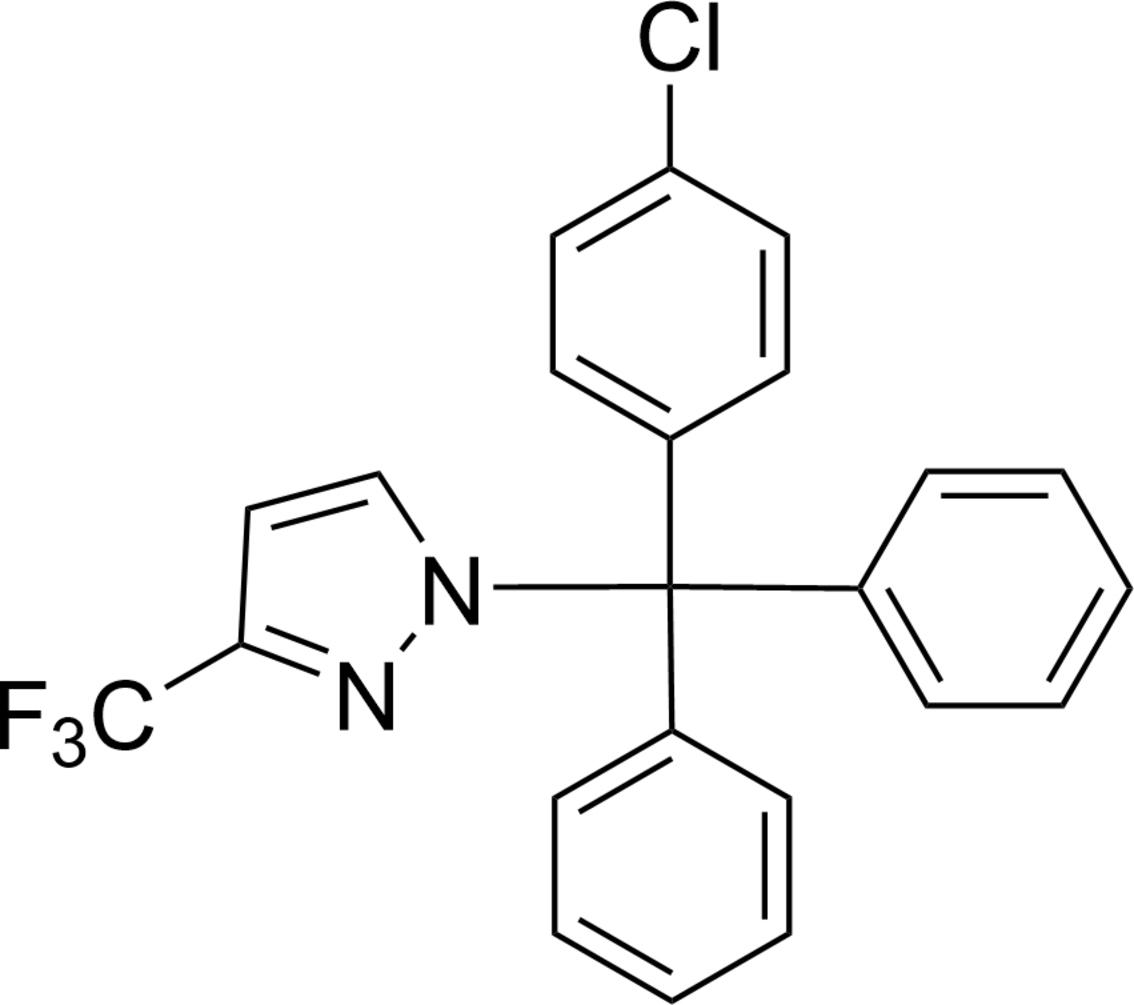


## 2. Structural commentary

**Figure 1:**
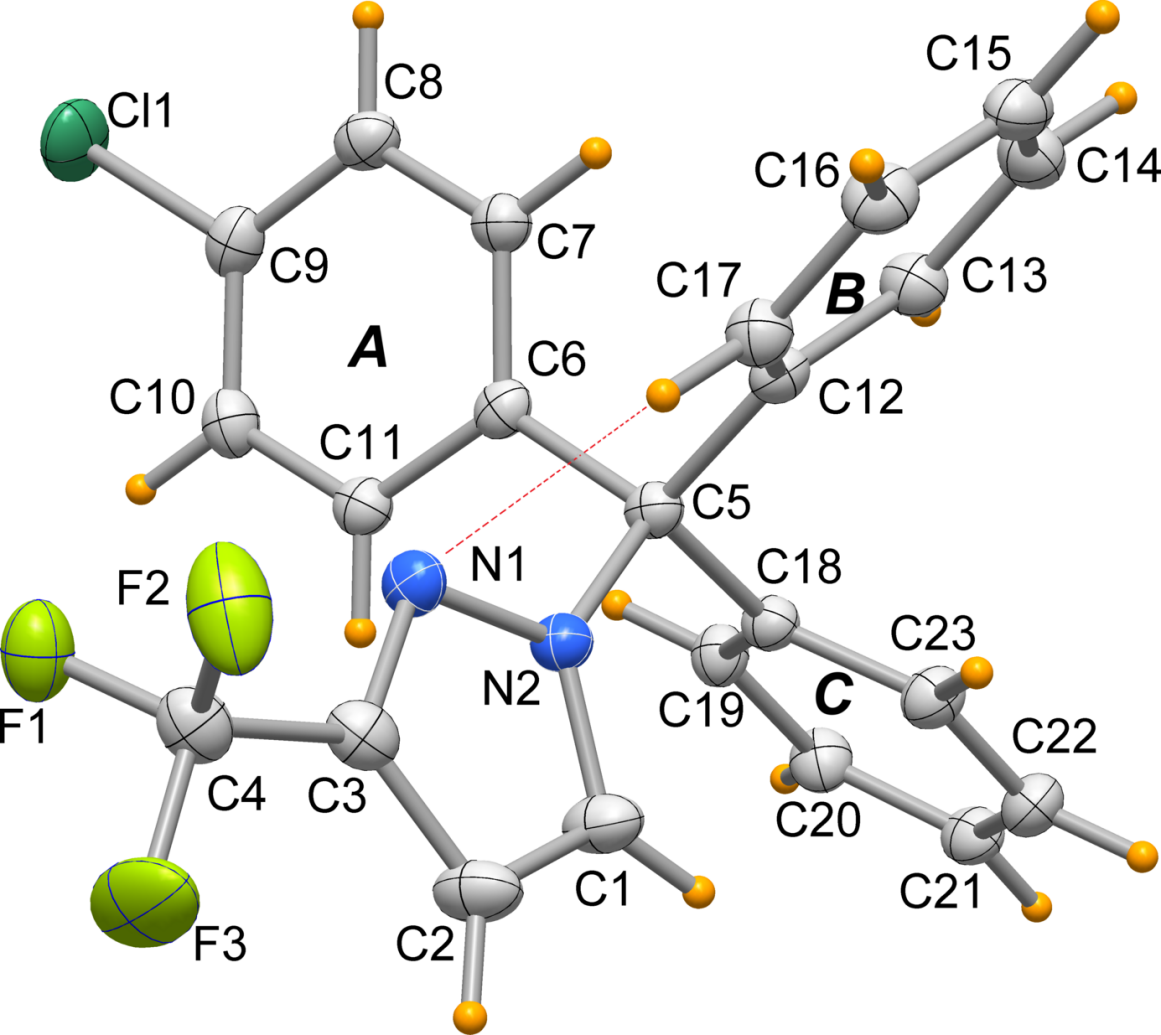
Mol­ecular structure of **1**. Displacement ellipsoids are drawn at the 50% probability level. The dotted line indicates the intra­molecular hydrogen bond.

The title mol­ecule, **1**, is shown in Fig. 1 ▸. The central carbon atom C5 exhibits a geometry close to ideal tetra­hedral and with similar C5—C(Ph) bond lengths (Table 1 ▸). The pyrazole ring geometry is unexceptional (Secrieru *et al.*, 2020 ▸). The phenyl rings show an irregular propeller conformation about the C5—N2 bond: the ring planes *A*, *B* and *C* are inclined to the C6/C12/C18 plane in the same sense by 43.15 (5), 70.58 (6) and 22.62 (6)°, respectively. These angles (φ) vary much more widely than in 1,1,1-tri­phenyl­ethane or tri­phenyl­chloro­methane (see Section 4), partly as a result of the presence of the pyrazole ring, which assumes a nearly eclipsed orientation with a C1—N2—C5—C18 torsion angle of 16.58 (18)°, and partly because of the intra­molecular C17—H17⋯N1 hydrogen bond (Table 2 ▸). It is noteworthy that the observed orientation of ring *B* (which shows the largest φ angle) is close to the simulated orientation (with φ = 64°) that would give the shortest H17⋯N1 distance. This can be seen as the proof that this contact is a stabilizing hydrogen bond, rather than an incidental effect of crystal packing.

## 3. Supra­molecular features

**Figure 2:**
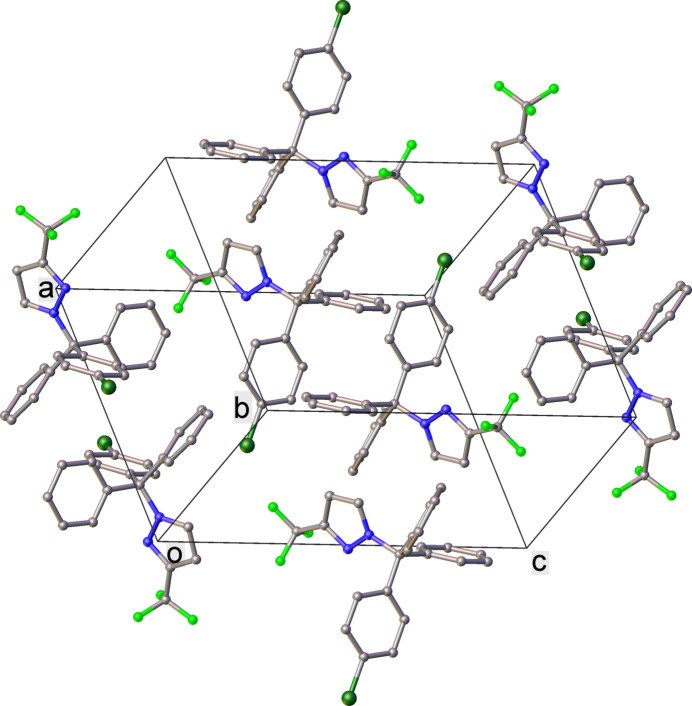
Crystal packing of **1** (H atoms are omitted for clarity).

**Figure 3:**
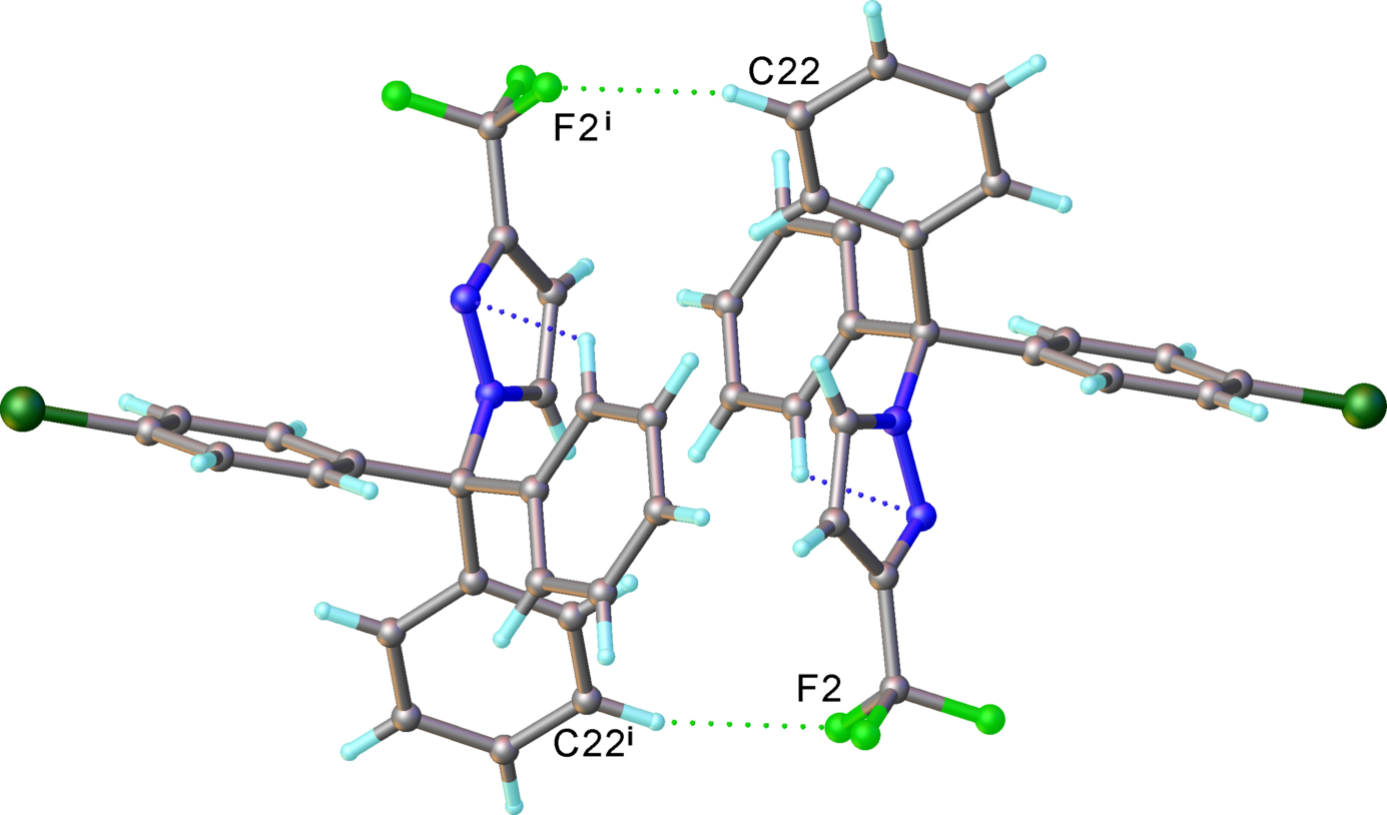
Inter­molecular hydrogen bonds in the structure of **1**. Symmetry code: (i) −*x* + 2, −*y* + 1, −*z* + 1.

The crystal packing is shown in Fig. 2 ▸. Mol­ecules are linked into centrosymmetric dimers by pairs of C22—H⋯F2 hydrogen bonds (Table 2 ▸, Fig. 3 ▸). Neither the phenyl nor the pyrazole rings are involved in π–π stacking inter­actions; however, there are C—H⋯π-type inter­actions between rings contacting edge-to-face, at inter­planar angles of 69.62 (6) to 78.68 (5)° and H⋯ring distances of 2.71–2.98 Å. Numerical details of hydoren bonds and C—H⋯π inter­actions are given in Table 2 ▸.

## 4. Database survey

The propeller conformation of the CPh_3_ moiety in **1** can be compared with those in 1,1,1-tri­phenyl­ethane (**2**) and tri­phenyl­chloro­methane (**3**). In **2**, the φ angles range from 41.3 to 55.3° at room temperature (TRPETN; Destro *et al.*, 1980 ▸) and from 42.0 to 53.9° at 100 K (TRPETN01; Fronczek, 2014 ▸). Three polymorphs of compound **3** have been reported: trigonal phase I and monoclinic phases II and III. Phase I (ZZZVTY12; Dunand & Gerdil, 1982 ▸) contains three crystallographically non-equivalent mol­ecules, each lying on a threefold axis and thus having a regular propeller conformation, with φ = 43.4, 47.0 and 51.0°. Phase II (ZZZVTY03; Kahr & Carter, 1992 ▸) has three mol­ecules per asymmetric unit, with φ varying from 38.2 to 59.0°, whereas phase III has five, with φ = 36.9–57.5° at 248 K (ZZZVTY04; Kahr & Carter, 1992 ▸) and φ = 34.6–58.2° at 100 K (ZZZVTY13; Wang *et al.*, 2013 ▸).

## 5. Synthesis and crystallization

A mixture of 435 mg (3.2 mmol) of 3-(tri­fluoro­meth­yl)-1*H*-pyrazole and 485 mg (3.5 mmol) of K_2_CO_3_ was dissolved in 20 mL of tetra­hydro­furan and stirred at reflux for 10–15 minutes. Then 1.00 g (3.2 mmol) of chloro­(4-chloro­phen­yl)methyl­ene)di­benzene was added to the reaction mixture and continued to boil for 5 h. After completion of the reaction, tetra­hydro­furan was removed under vacuum and 10 mL of diethyl ether were added to the obtained oily residue, which formed compound **1** as a solid product. Colourless prismatic crystals suitable for X-ray analysis were obtained by slow evaporation of an aceto­nitrile solution.

Yield: 936 mg (71%); m.p. 355–360 K. Analysis calculated (%) for C_23_H_16_ClF_3_N_2_: C 66.92, H 3.91, N 6.79; found C 66.90, H 3.90, N 6.77. ^1^H NMR (300 MHz, CDCl_3_): 6.52–6.53 (1H, CF_3_CCH), 7.07–7.37 (10H, 2Ph, 4H, 4-ClPh, 1H, NCH). ^13^C NMR (75 MHz, CDCl_3_): 79.24, 103.19, 111.07, 127.93, 128.04, 128.30, 130.01, 131.65, 133.79, 134.05, 139.12, 141.17, 142.18. ESI-MS: 413.8 (*M* + H^+^).

## 6. Refinement

Crystal data, data collection and structure refinement details are summarized in Table 3 ▸. H atoms were placed geometrically with C—H = 0.95 Å and included in the refinement in the riding-motion model with *U*_iso_(H) = 1.2*U*_eq_(C), except H17 and H22 which were refined in an isotropic approximation. About 50 hkl data were missed due to collection *via* the spindle axis only.

## Supplementary Material

Crystal structure: contains datablock(s) I. DOI: 10.1107/S2056989025001185/zv2037sup1.cif

Structure factors: contains datablock(s) I. DOI: 10.1107/S2056989025001185/zv2037Isup2.hkl

Supporting information file. DOI: 10.1107/S2056989025001185/zv2037Isup3.cml

CCDC reference: 2422692

Additional supporting information:  crystallographic information; 3D view; checkCIF report

## Figures and Tables

**Table 1:** Selected geometric parameters (Å, °)

N1—N2	1.3517 (16)	C1—C2	1.373 (2)
N1—C3	1.3328 (19)	C5—C6	1.5400 (18)
N2—C1	1.3575 (19)	C5—C12	1.5431 (18)
N2—C5	1.4981 (17)	C5—C18	1.5511 (17)
			
N2—C5—C6	106.37 (10)	C6—C5—C12	110.99 (11)
N2—C5—C12	110.49 (10)	C6—C5—C18	112.58 (10)
N2—C5—C18	107.06 (10)	C12—C5—C18	109.24 (10)

**Table 2:** Hydrogen-bond geometry and C—H⋯π interaction parameters (Å, °)

*D*—H⋯*A*	*D*—H	H⋯*A*	*D*⋯*A*	*D*—H⋯*A*
C17—H17⋯N1	0.98 (2)	2.382 (19)	3.0541 (19)	125.1 (14)
C22—H22⋯F2^i^	0.96 (2)	2.48 (2)	3.3596 (18)	151.6 (16)
C2—H2⋯*Cg*1^i^	0.95	2.96	3.6237 (17)	128
C7—H7⋯*Cg*1	0.95	2.98	3.6784 (15)	132
C8—H8⋯*Cg*2^ii^	0.95	2.80	3.4721 (15)	129
C13—H13⋯*Cg*3^ii^	0.95	2.98	3.7873 (15)	144
C21—H21⋯*Cg*3^iii^	0.95	2.71	3.4928 (15)	140

Symmetry codes: (i) 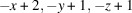; (ii) 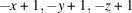; (iii) 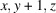.

**Table 3:** Experimental details

Crystal data	
Chemical formula	C_23_H_16_ClF_3_N_2_
*M*_r_	412.83
Crystal system, space group	Monoclinic, *P*2_1_/*c*
Temperature (K)	100
*a*, *b*, *c* (Å)	12.12054 (10), 8.93314 (6), 17.75198 (15)
β (°)	90.4278 (8)
*V* (Å^3^)	1922.03 (3)
*Z*	4
Radiation type	Cu *K*α
μ (mm^−1^)	2.11
Crystal size (mm)	0.18 × 0.11 × 0.06

Data collection	
Diffractometer	XtaLAB Synergy, Dualflex, HyPix
Absorption correction	Gaussian (*CrysAlis PRO*; Rigaku OD, 2024 ▸)
*T*_min_, *T*_max_	0.688, 1.000
No. of measured, independent and observed [*I* > 2σ(*I*)] reflections	25542, 4102, 3917
*R*_int_	0.026
(sin θ/λ)_max_ (Å^−1^)	0.638

Refinement	
*R*[*F*^2^ > 2σ(*F*^2^)], *wR*(*F*^2^), *S*	0.038, 0.108, 1.07
No. of reflections	4102
No. of parameters	270
H-atom treatment	H atoms treated by a mixture of independent and constrained refinement
Δρ_max_, Δρ_min_ (e Å^−3^)	0.44, −0.46

Computer programs: *CrysAlis PRO* (Rigaku OD, 2024 ▸), *SHELXT2014/5* (Sheldrick, 2015*a* ▸), *SHELXL2019/2* (Sheldrick, 2015*b* ▸), *DIAMOND* (Brandenburg & Putz, 1999 ▸ and *Crystal Explorer 17.5* (Spackman *et al.*, 2021 ▸).

## supplementary crystallographic information

### 1-[(4-Chlorophenyl)diphenylmethyl]-3-(trifluoromethyl)-1H-pyrazole Crystal data

**Table:**

C_23_H_16_ClF_3_N_2_	*F*(000) = 848
*M**_r_* = 412.83	*D*_x_ = 1.427 Mg m^−^^3^
Monoclinic, *P*2_1_/*c*	Cu *K*α radiation, λ = 1.54184 Å
*a* = 12.12054 (10) Å	Cell parameters from 19127 reflections
*b* = 8.93314 (6) Å	θ = 2.5–79.5°
*c* = 17.75198 (15) Å	µ = 2.11 mm^−^^1^
β = 90.4278 (8)°	*T* = 100 K
*V* = 1922.03 (3) Å^3^	Prism, colorless
*Z* = 4	0.18 × 0.11 × 0.06 mm

### 1-[(4-Chlorophenyl)diphenylmethyl]-3-(trifluoromethyl)-1H-pyrazole Data collection

**Table:**

XtaLAB Synergy, Dualflex, HyPix diffractometer	3917 reflections with *I* > 2σ(*I*)
Radiation source: micro-focus sealed X-ray tube	*R*_int_ = 0.026
ω scans	θ_max_ = 79.8°, θ_min_ = 3.7°
Absorption correction: gaussian (CrysAlisPro; Rigaku OD, 2024)	*h* = −14→15
*T*_min_ = 0.688, *T*_max_ = 1.000	*k* = −10→11
25542 measured reflections	*l* = −22→22
4102 independent reflections	

### 1-[(4-Chlorophenyl)diphenylmethyl]-3-(trifluoromethyl)-1H-pyrazole Refinement

**Table:**

Refinement on *F*^2^	Primary atom site location: dual
Least-squares matrix: full	Secondary atom site location: difference Fourier map
*R*[*F*^2^ > 2σ(*F*^2^)] = 0.038	Hydrogen site location: inferred from neighbouring sites
*wR*(*F*^2^) = 0.108	H atoms treated by a mixture of independent and constrained refinement
*S* = 1.07	*w* = 1/[σ^2^(*F*_o_^2^) + (0.056*P*)^2^ + 0.9694*P*] where *P* = (*F*_o_^2^ + 2*F*_c_^2^)/3
4102 reflections	(Δ/σ)_max_ = 0.001
270 parameters	Δρ_max_ = 0.44 e Å^−^^3^
0 restraints	Δρ_min_ = −0.46 e Å^−^^3^

### 1-[(4-Chlorophenyl)diphenylmethyl]-3-(trifluoromethyl)-1H-pyrazole Special details

**Table:**

Geometry. All esds (except the esd in the dihedral angle between two l.s. planes) are estimated using the full covariance matrix. The cell esds are taken into account individually in the estimation of esds in distances, angles and torsion angles; correlations between esds in cell parameters are only used when they are defined by crystal symmetry. An approximate (isotropic) treatment of cell esds is used for estimating esds involving l.s. planes.

### 1-[(4-Chlorophenyl)diphenylmethyl]-3-(trifluoromethyl)-1H-pyrazole Fractional atomic coordinates and isotropic or equivalent isotropic displacement parameters (Å^2^)

**Table:**

	*x*	*y*	*z*	*U*_iso_*/*U*_eq_	
Cl1	0.36774 (3)	0.04033 (5)	0.33222 (2)	0.03673 (13)	
F1	0.93996 (8)	−0.01654 (11)	0.32090 (6)	0.0377 (2)	
F2	1.04152 (11)	0.00747 (12)	0.41780 (6)	0.0499 (3)	
F3	1.09493 (10)	0.09519 (13)	0.31067 (8)	0.0583 (4)	
N1	0.86043 (10)	0.23237 (13)	0.40816 (7)	0.0227 (2)	
N2	0.83992 (9)	0.38039 (12)	0.41539 (6)	0.0201 (2)	
C1	0.92135 (13)	0.46447 (17)	0.38471 (9)	0.0290 (3)	
H1	0.924180	0.570700	0.383099	0.035*	
C2	0.99888 (13)	0.36792 (18)	0.35644 (9)	0.0320 (3)	
H2	1.065849	0.391975	0.331748	0.038*	
C3	0.95659 (12)	0.22665 (17)	0.37235 (8)	0.0244 (3)	
C4	1.00804 (12)	0.07923 (18)	0.35536 (9)	0.0280 (3)	
C5	0.73785 (11)	0.42950 (14)	0.45553 (7)	0.0190 (3)	
C6	0.64342 (11)	0.32991 (14)	0.42577 (8)	0.0192 (3)	
C7	0.56995 (11)	0.25784 (15)	0.47346 (8)	0.0213 (3)	
H7	0.577978	0.269227	0.526420	0.026*	
C8	0.48472 (12)	0.16921 (16)	0.44485 (8)	0.0239 (3)	
H8	0.434854	0.120859	0.477957	0.029*	
C9	0.47364 (12)	0.15249 (16)	0.36766 (8)	0.0245 (3)	
C10	0.54608 (12)	0.22183 (15)	0.31846 (8)	0.0239 (3)	
H10	0.537938	0.209519	0.265557	0.029*	
C11	0.63059 (11)	0.30946 (14)	0.34793 (8)	0.0210 (3)	
H11	0.680799	0.356557	0.314612	0.025*	
C12	0.75251 (11)	0.41134 (14)	0.54147 (7)	0.0198 (3)	
C13	0.68273 (12)	0.49156 (16)	0.58885 (8)	0.0242 (3)	
H13	0.629103	0.556908	0.567554	0.029*	
C14	0.69039 (13)	0.47746 (17)	0.66662 (8)	0.0267 (3)	
H14	0.642528	0.533415	0.698044	0.032*	
C15	0.76794 (13)	0.38167 (16)	0.69848 (8)	0.0261 (3)	
H15	0.773672	0.371902	0.751657	0.031*	
C16	0.83678 (13)	0.30059 (16)	0.65192 (8)	0.0268 (3)	
H16	0.889550	0.234290	0.673460	0.032*	
C17	0.82977 (12)	0.31486 (16)	0.57365 (8)	0.0239 (3)	
H17	0.8794 (16)	0.257 (2)	0.5412 (11)	0.028 (4)*	
C18	0.72023 (11)	0.59740 (14)	0.43669 (7)	0.0197 (3)	
C19	0.64102 (12)	0.64777 (15)	0.38557 (8)	0.0225 (3)	
H19	0.591919	0.578336	0.362534	0.027*	
C20	0.63317 (12)	0.80002 (16)	0.36787 (8)	0.0248 (3)	
H20	0.578844	0.833085	0.332791	0.030*	
C21	0.70372 (13)	0.90285 (15)	0.40092 (8)	0.0247 (3)	
H21	0.699169	1.005792	0.387826	0.030*	
C22	0.78131 (12)	0.85427 (16)	0.45345 (8)	0.0256 (3)	
H22	0.8306 (17)	0.924 (2)	0.4781 (11)	0.031 (5)*	
C23	0.78900 (12)	0.70327 (16)	0.47162 (8)	0.0237 (3)	
H23	0.841568	0.671365	0.508187	0.028*	

### 1-[(4-Chlorophenyl)diphenylmethyl]-3-(trifluoromethyl)-1H-pyrazole Atomic displacement parameters (Å^2^)

**Table:**

	*U*^11^	*U*^22^	*U*^33^	*U*^12^	*U*^13^	*U*^23^
Cl1	0.0276 (2)	0.0408 (2)	0.0417 (2)	−0.00851 (14)	−0.00535 (16)	−0.01070 (16)
F1	0.0353 (5)	0.0348 (5)	0.0429 (5)	0.0067 (4)	−0.0064 (4)	−0.0102 (4)
F2	0.0671 (8)	0.0418 (6)	0.0404 (6)	0.0279 (5)	−0.0212 (5)	−0.0061 (5)
F3	0.0413 (6)	0.0447 (6)	0.0894 (9)	0.0068 (5)	0.0370 (6)	−0.0065 (6)
N1	0.0209 (6)	0.0201 (5)	0.0270 (6)	0.0029 (4)	0.0014 (4)	−0.0007 (4)
N2	0.0195 (5)	0.0188 (5)	0.0219 (5)	0.0014 (4)	0.0009 (4)	0.0017 (4)
C1	0.0264 (7)	0.0245 (7)	0.0363 (8)	−0.0001 (5)	0.0081 (6)	0.0066 (6)
C2	0.0256 (7)	0.0317 (8)	0.0390 (8)	0.0017 (6)	0.0107 (6)	0.0070 (6)
C3	0.0210 (6)	0.0297 (7)	0.0224 (6)	0.0037 (5)	−0.0006 (5)	0.0011 (5)
C4	0.0227 (7)	0.0322 (8)	0.0290 (7)	0.0053 (6)	0.0007 (6)	−0.0013 (6)
C5	0.0184 (6)	0.0176 (6)	0.0211 (6)	0.0013 (5)	0.0012 (5)	0.0011 (5)
C6	0.0185 (6)	0.0149 (5)	0.0241 (6)	0.0029 (4)	−0.0003 (5)	0.0009 (5)
C7	0.0210 (6)	0.0203 (6)	0.0228 (6)	0.0017 (5)	0.0010 (5)	0.0006 (5)
C8	0.0205 (6)	0.0219 (6)	0.0294 (7)	0.0004 (5)	0.0035 (5)	0.0011 (5)
C9	0.0198 (6)	0.0213 (6)	0.0322 (7)	0.0024 (5)	−0.0031 (5)	−0.0035 (5)
C10	0.0256 (7)	0.0229 (6)	0.0230 (6)	0.0053 (5)	−0.0030 (5)	−0.0018 (5)
C11	0.0225 (6)	0.0179 (6)	0.0225 (6)	0.0033 (5)	0.0006 (5)	0.0020 (5)
C12	0.0197 (6)	0.0180 (6)	0.0217 (6)	−0.0022 (5)	−0.0006 (5)	0.0020 (5)
C13	0.0246 (7)	0.0238 (6)	0.0242 (7)	0.0026 (5)	0.0003 (5)	0.0022 (5)
C14	0.0278 (7)	0.0278 (7)	0.0246 (7)	0.0011 (6)	0.0030 (6)	−0.0007 (5)
C15	0.0295 (7)	0.0272 (7)	0.0216 (6)	−0.0058 (6)	−0.0016 (5)	0.0034 (5)
C16	0.0286 (7)	0.0247 (7)	0.0270 (7)	0.0017 (5)	−0.0044 (6)	0.0055 (5)
C17	0.0250 (7)	0.0218 (6)	0.0249 (7)	0.0019 (5)	−0.0012 (5)	0.0013 (5)
C18	0.0207 (6)	0.0174 (6)	0.0210 (6)	0.0010 (5)	0.0017 (5)	0.0014 (5)
C19	0.0245 (6)	0.0197 (6)	0.0231 (6)	0.0012 (5)	−0.0013 (5)	−0.0004 (5)
C20	0.0288 (7)	0.0211 (6)	0.0245 (6)	0.0037 (5)	−0.0024 (5)	0.0027 (5)
C21	0.0296 (7)	0.0177 (6)	0.0269 (7)	0.0012 (5)	0.0038 (6)	0.0030 (5)
C22	0.0272 (7)	0.0211 (6)	0.0284 (7)	−0.0042 (5)	0.0005 (6)	−0.0006 (5)
C23	0.0237 (7)	0.0218 (6)	0.0255 (6)	−0.0013 (5)	−0.0019 (5)	0.0021 (5)

### 1-[(4-Chlorophenyl)diphenylmethyl]-3-(trifluoromethyl)-1H-pyrazole Geometric parameters (Å, º)

**Table:**

Cl1—C9	1.7422 (14)	C10—C11	1.388 (2)
F1—C4	1.3340 (18)	C11—H11	0.9500
F2—C4	1.3408 (18)	C12—C13	1.395 (2)
F3—C4	1.3308 (19)	C12—C17	1.3920 (19)
N1—N2	1.3517 (16)	C13—H13	0.9500
N1—C3	1.3328 (19)	C13—C14	1.389 (2)
N2—C1	1.3575 (19)	C14—H14	0.9500
N2—C5	1.4981 (17)	C14—C15	1.388 (2)
C1—H1	0.9500	C15—H15	0.9500
C1—C2	1.373 (2)	C15—C16	1.384 (2)
C2—H2	0.9500	C16—H16	0.9500
C2—C3	1.392 (2)	C16—C17	1.397 (2)
C3—C4	1.489 (2)	C17—H17	0.98 (2)
C5—C6	1.5400 (18)	C18—C19	1.3910 (19)
C5—C12	1.5431 (18)	C18—C23	1.4021 (19)
C5—C18	1.5511 (17)	C19—H19	0.9500
C6—C7	1.3911 (19)	C19—C20	1.3990 (19)
C6—C11	1.4013 (18)	C20—H20	0.9500
C7—H7	0.9500	C20—C21	1.383 (2)
C7—C8	1.394 (2)	C21—H21	0.9500
C8—H8	0.9500	C21—C22	1.389 (2)
C8—C9	1.384 (2)	C22—H22	0.96 (2)
C9—C10	1.389 (2)	C22—C23	1.390 (2)
C10—H10	0.9500	C23—H23	0.9500
			
C3—N1—N2	104.17 (11)	C6—C11—H11	119.3
N1—N2—C1	111.62 (12)	C10—C11—C6	121.41 (13)
N1—N2—C5	118.99 (11)	C10—C11—H11	119.3
C1—N2—C5	129.37 (12)	C13—C12—C5	118.43 (12)
N2—C1—H1	126.3	C17—C12—C5	122.88 (12)
N2—C1—C2	107.50 (13)	C17—C12—C13	118.66 (12)
C2—C1—H1	126.3	C12—C13—H13	119.5
C1—C2—H2	128.0	C14—C13—C12	121.08 (13)
C1—C2—C3	103.96 (13)	C14—C13—H13	119.5
C3—C2—H2	128.0	C13—C14—H14	120.0
N1—C3—C2	112.75 (13)	C15—C14—C13	120.06 (14)
N1—C3—C4	119.98 (13)	C15—C14—H14	120.0
C2—C3—C4	127.26 (14)	C14—C15—H15	120.4
F1—C4—F2	104.77 (13)	C16—C15—C14	119.25 (13)
F1—C4—C3	113.68 (12)	C16—C15—H15	120.4
F2—C4—C3	112.33 (12)	C15—C16—H16	119.5
F3—C4—F1	106.56 (13)	C15—C16—C17	120.92 (13)
F3—C4—F2	107.94 (13)	C17—C16—H16	119.5
F3—C4—C3	111.13 (13)	C12—C17—C16	120.02 (14)
N2—C5—C6	106.37 (10)	C12—C17—H17	119.9 (11)
N2—C5—C12	110.49 (10)	C16—C17—H17	120.1 (11)
N2—C5—C18	107.06 (10)	C19—C18—C5	123.14 (12)
C6—C5—C12	110.99 (11)	C19—C18—C23	118.39 (12)
C6—C5—C18	112.58 (10)	C23—C18—C5	118.46 (11)
C12—C5—C18	109.24 (10)	C18—C19—H19	119.8
C7—C6—C5	122.41 (12)	C18—C19—C20	120.43 (13)
C7—C6—C11	118.22 (12)	C20—C19—H19	119.8
C11—C6—C5	119.36 (12)	C19—C20—H20	119.7
C6—C7—H7	119.4	C21—C20—C19	120.65 (13)
C6—C7—C8	121.14 (13)	C21—C20—H20	119.7
C8—C7—H7	119.4	C20—C21—H21	120.3
C7—C8—H8	120.4	C20—C21—C22	119.39 (13)
C9—C8—C7	119.21 (13)	C22—C21—H21	120.3
C9—C8—H8	120.4	C21—C22—H22	121.3 (12)
C8—C9—Cl1	119.04 (12)	C21—C22—C23	120.19 (13)
C8—C9—C10	121.16 (13)	C23—C22—H22	118.6 (12)
C10—C9—Cl1	119.80 (11)	C18—C23—H23	119.6
C9—C10—H10	120.6	C22—C23—C18	120.90 (13)
C11—C10—C9	118.86 (13)	C22—C23—H23	119.6
C11—C10—H10	120.6		
			
Cl1—C9—C10—C11	−179.33 (10)	C5—C18—C23—C22	176.11 (13)
N1—N2—C1—C2	−0.28 (18)	C6—C5—C12—C13	−81.65 (15)
N1—N2—C5—C6	−44.59 (14)	C6—C5—C12—C17	96.19 (15)
N1—N2—C5—C12	75.97 (14)	C6—C5—C18—C19	−12.64 (18)
N1—N2—C5—C18	−165.17 (11)	C6—C5—C18—C23	168.79 (12)
N1—C3—C4—F1	51.16 (19)	C6—C7—C8—C9	0.3 (2)
N1—C3—C4—F2	−67.59 (18)	C7—C6—C11—C10	1.02 (19)
N1—C3—C4—F3	171.36 (14)	C7—C8—C9—Cl1	179.45 (10)
N2—N1—C3—C2	0.17 (16)	C7—C8—C9—C10	0.3 (2)
N2—N1—C3—C4	178.79 (12)	C8—C9—C10—C11	−0.2 (2)
N2—C1—C2—C3	0.35 (18)	C9—C10—C11—C6	−0.5 (2)
N2—C5—C6—C7	131.58 (12)	C11—C6—C7—C8	−0.89 (19)
N2—C5—C6—C11	−48.00 (15)	C12—C5—C6—C7	11.35 (17)
N2—C5—C12—C13	160.59 (12)	C12—C5—C6—C11	−168.23 (11)
N2—C5—C12—C17	−21.57 (17)	C12—C5—C18—C19	−136.42 (13)
N2—C5—C18—C19	103.91 (14)	C12—C5—C18—C23	45.01 (16)
N2—C5—C18—C23	−74.66 (14)	C12—C13—C14—C15	−0.4 (2)
C1—N2—C5—C6	137.16 (14)	C13—C12—C17—C16	−0.4 (2)
C1—N2—C5—C12	−102.28 (16)	C13—C14—C15—C16	−0.2 (2)
C1—N2—C5—C18	16.58 (18)	C14—C15—C16—C17	0.5 (2)
C1—C2—C3—N1	−0.33 (18)	C15—C16—C17—C12	−0.2 (2)
C1—C2—C3—C4	−178.83 (14)	C17—C12—C13—C14	0.7 (2)
C2—C3—C4—F1	−130.44 (16)	C18—C5—C6—C7	−111.46 (14)
C2—C3—C4—F2	110.81 (18)	C18—C5—C6—C11	68.96 (15)
C2—C3—C4—F3	−10.2 (2)	C18—C5—C12—C13	43.06 (16)
C3—N1—N2—C1	0.07 (15)	C18—C5—C12—C17	−139.10 (13)
C3—N1—N2—C5	−178.47 (11)	C18—C19—C20—C21	−0.1 (2)
C5—N2—C1—C2	178.07 (13)	C19—C18—C23—C22	−2.5 (2)
C5—C6—C7—C8	179.53 (12)	C19—C20—C21—C22	−1.5 (2)
C5—C6—C11—C10	−179.39 (12)	C20—C21—C22—C23	1.0 (2)
C5—C12—C13—C14	178.64 (13)	C21—C22—C23—C18	1.0 (2)
C5—C12—C17—C16	−178.24 (13)	C23—C18—C19—C20	2.1 (2)
C5—C18—C19—C20	−176.47 (13)		

### 1-[(4-Chlorophenyl)diphenylmethyl]-3-(trifluoromethyl)-1H-pyrazole Hydrogen-bond geometry (Å, º)

*Cg*1, *Cg*2 and *Cg*3 are the centroids of the C12–17, C18–23 and C6–11 rings, respectively.

**Table:**

*D*—H···*A*	*D*—H	H···*A*	*D*···*A*	*D*—H···*A*
C17—H17···N1	0.98 (2)	2.382 (19)	3.0541 (19)	125.1 (14)
C22—H22···F2^i^	0.96 (2)	2.48 (2)	3.3596 (18)	151.6 (16)
C2—H2···*Cg*1^i^	0.95	2.96	3.6237 (17)	128
C7—H7···*Cg*1	0.95	2.98	3.6784 (15)	132
C8—H8···*Cg*2^ii^	0.95	2.80	3.4721 (15)	129
C13—H13···*Cg*3^ii^	0.95	2.98	3.7873 (15)	144
C21—H21···*Cg*3^iii^	0.95	2.71	3.4928 (15)	140

Symmetry codes: (i) −*x*+2, −*y*+1, −*z*+1; (ii) −*x*+1, −*y*+1, −*z*+1; (iii) *x*, *y*+1, *z*.
